# A palliative care link nurse programme in Mulago Hospital, Uganda: an evaluation using mixed methods

**DOI:** 10.1186/s12904-016-0115-6

**Published:** 2016-04-08

**Authors:** Julia Downing, Mwazi Batuli, Grace Kivumbi, Josephine Kabahweza, Liz Grant, Scott A. Murray, Elizabeth Namukwaya, Mhoira Leng

**Affiliations:** Makerere University, PO Box 7757, Kampala, Uganda; Makerere Palliative Care Unit, PO Box 7757, Kampala, Uganda; Mulago Hospital, PO Box 7757, Kampala, Uganda; Global Health Academy, Centre for Population Health Sciences, University of Edinburgh. Medical School, Teviot Place, Edinburgh, EH8 9AG UK; St. Columba’s Hospice Chair of Primary Palliative Care, Primary Palliative Care Research Group, University of Edinburgh, Medical School, Teviot Place, Edinburgh, EH8 9AG UK

**Keywords:** Palliative care, Hospital, Uganda, Africa, Service delivery, Education, Evaluation, Nurses, Generalist palliative care, Mixed methods

## Abstract

**Background:**

Integrating palliative care (PC) and empowering the health care workforce is essential to achieve universal access to PC services. In 2010, 46 % of patients in Mulago Hospital, Uganda had a life limiting illness, of whom 96 % had PC needs. The university/hospital specialist PC unit (Makerere/Mulago Palliative Care Unit –MPCU) implemented a link-nurse model to empower hospital nurses to provide generalist PC. Over two years, 27 link nurses were trained and mentored and 11 clinical protocols developed. The aim of the study was to evaluate the impact of the palliative care link nurse programme at Mulago Hospital

**Methods:**

An evaluation approach utilising mixed methods was used integrating qualitative and quantitative data including: pre and post course assessment confidence ratings; course evaluation forms; audit of clinical guidelines availability; review of link-nurse activity sheets/action plans; review of MPCU patient documentation; Most Significant Change (MSC); individual and focus group interviews.

**Results:**

A significant difference was seen in nurses’ confidence after the training (*p* < 0.001). From July 2012 to December 2013, link nurses identified 2447 patients needing PC, of whom they cared for 2113 (86 %) and referred 334 (14 %) to MPCU. Clinical guidelines/protocols were utilised in 50 % of wards. Main themes identified include: change in attitude; developing new skills and knowledge; change in relationships; improved outcomes of care, along with the challenges that they experienced in integrating PC. Since the start of the programme there has been an increase in PC patients seen at the hospital (611 in 2011 to 1788 in 2013).

**Conclusion:**

The link-nurse programme is a practical model for integrating PC into generalist services. Recommendations have been made for ongoing development and expansion of the programme as an effective health systems strengthening approach in similar healthcare contexts, as well as the improvement in medical and nursing education.

## Background

Palliative care is still absent or severely limited in many parts of the world, including Africa, where a continuing effort to expand the scope and quality of palliative care is needed. Access to culturally appropriate holistic palliative care is often limited, and at worst non-existent, [1] although there has been progress in the last decade [2]. Palliative care is a global concern [3, 4] and a public health approach is central to the development of palliative care in Africa and models of palliative care delivery must be developed and adapted to meet the unique needs of people with all life-threatening illnesses in different cultures, communities and countries, whilst ensuring that it is provided at primary, secondary and tertiary levels of care [5, 6].

Palliative care i.e. an approach that improves the quality of life of individuals and their families facing the problem associated with life-threatening illness [7]; should be offered as an essential service based on need and not limited by diagnosis, setting, economic constraints or cultural context. Integration of services into existing health systems is important in ensuring both accessibility and sustainability of services and is a key component of the World Health Assembly (WHA) resolution, signed in 2014 [8]. Hospital based palliative care in Africa is less well developed than care in the community setting where most of the population will live and die. However, many patients are hospitalised during initial diagnosis or when receiving treatment before they are referred on for community based care [9]. Integrated hospital based services, as part of a continuum of care with the community, provides an opportunity to start palliative care earlier in the patient’s disease trajectory, implement referral pathways and influence the overall health system [9].

Mulago Hospital is Uganda’s National Referral and Teaching Hospital with 1790 beds and is located on the Mulago site alongside the Uganda Heart Institute (UHI) and Uganda Cancer Institute (UCI) [10]. It provides specialist services admitting over 140,000 patients and with 600,000 out-patients annually. A needs assessment in 2009 revealed 46 % of admissions had life-limiting illness. High levels of symptoms (pain 70 %, weakness 87 %, and cough 62 %) were identified, along with significant social, psychological and spiritual distress. Only 5 % of these patients were accessing the palliative care service [11]. The Palliative Care Unit (MPCU) was established in Mulago National Referral Hospital in 2006 and joined with Makerere University in 2008. The team offers clinical care across the Mulago site and includes physicians, training grade doctors, specialist palliative care nurses and clinical officer, an honorary nursing professor, a social and pastoral care co-ordinator with trained volunteers and administrators. Services are operated as consultancy services seeing patients jointly with other hospital teams, thus providing specialist palliative care support alongside generalist palliative care (i.e. palliative care provided as an integral part of standard clinical practice by any healthcare professional who is not part of a specialist palliative care team [12]). Alongside this, the MPCU teaches and learns from other specialists in the hospital and has established a palliative research agenda and an active programme of undergraduate and post graduate training. During 2011 the team looked after 643 patients and families within Mulago Hospital [9]. With almost half of inpatients having some palliative care need, it is not possible for the team to see all of them. Thus the link-nurse programme was initiated to support MPCU to develop an integrated model ensuring palliative care is available at both the generalist and specialist levels and available to all in need.

‘Link-nurses’ were first introduced in the UK in 1988 in the area of infection control [13]. Since then they have been used in colorectal care [14], tissue viability [15], diabetes [16], continence [17], nursing research and development [18] and palliative care [19–23], amongst others. They have also been used across different settings, such as within acute hospitals [22], primary care [24] and nursing/care homes [20, 21, 25]. Whilst the model of link nurses has changed and over the years there has been little consensus on the most effective model [26], the core remit has remained the same i.e. “a ‘link nurse’ is a named individual who, supported by other practitioners, would act as a local resource and disseminate information to their colleagues” (p805) [25]. They are practising nurses with an expressed interest in a specialty such as palliative care, with formal links to specialist staff such as clinical nurse specialists [16, 21], and in this case the MPCU. Whilst there is literature available on link nurses in palliative care from the UK, this is the first time that it has been implemented in a setting such as Mulago Hospital in Uganda.

The link-nurse programme (Table 1) was initially run for a 2-year period commencing in 2011, through a grant from the Diana Princess of Wales Memorial Fund (URN 6596/3201), and in collaboration with the University of Edinburgh. An evaluation of the programme was undertaken prior to expansion of the programme. The aim of the evaluation was to address the question “What has been the impact of the palliative care link-nurse programme implemented in Mulago Hospital and what are the recommendations for future service provision?”.

**Table 1:** The link-nurse programme

Aims of the link-nurse programme	• Improving palliative care provision within the Hospital. • Improving links between the specialist unit and the wards. • Equipping nurses from different units with knowledge and skills that will enable them to provide basic palliative care to their patients.
Components of the link-nurse programme	• The identification of nurses to be trained as link-nurses - with the help of the in-charges of the wards and the senior nursing management, link-nurses were selected from wards where there are a high proportion of patients in need of palliative care and individuals in senior clinical roles, who showed an interest in palliative care. • Training of the nurses in palliative care using an adapted 5 day training developed by MPCU and based on the palliative care toolkit [39] and training manual [40]. Nurses attended an initial 3-day training, followed by mentorship and support supervision on the wards by the link-nurse co-ordinator and a further 2-day training three months after the initial training. The training was based on an interactive format and involved lectures, case discussions, group work and role-play. • Provision of ongoing mentorship and support. • Implementation of the use of clinical guidelines developed by MPCU - for the second cohort the clinical protocols also formed a core part of the training, • Implementation of a ‘categorisation’ system to enable the MPCU team and the link-nurses to identify patients who can be supported by the link-nurses and those that need referral to the palliative care team. • Recording of basic information about the patients for whom the link-nurses provide palliative care. • Evaluation and improvement of the programme.

## Methods

A mixed methods approach was utilised in the evaluation. Evaluations are used to assess the worth or value of something and there is value in utilising both quantitative and qualitative data collection methods within the evaluation design in order to answer different questions within the overall evaluation [27]. Quantitative data was used to demonstrate the number of patients seen by link-nurses, improvement in knowledge and confidence of link-nurses. Qualitative data was used to understand the experiences of the link nurses, and provide examples of the impact of the link-nurse programme through thematic analysis, and has been reported according to the COREQ guidelines [28].

### Quantitative data collection methods

1. *Pre and post course confidence ratings* – link-nurses completed a pre and post course confidence rating during the taught component of the training.
2. *Course evaluation forms* – At the end of each training component the link nurses completed an evaluation form.
3. *Review of action plans* – the link nurses developed action plans as to how they were going to integrate what they have learnt, into their places of work.
4. *Audit re the availability of the clinical guidelines of the clinical guidelines and their utilisation*
5. *Review of activity database from the link-nurses and MPCU*

### Qualitative data collection methods

6. *Focus group discussions (FGDs) with the link-nurses and the MPCU team*
7. *Most Significant Change –* Link-nurses were asked to identify areas of most significant change that they had identified in three areas – for themselves, for the patients and for colleagues.

A summary of the data collection methods is presented in Table 2.

**Table 2:** Summary of data collection

Data	Data source	Participants		Time frame
1. *Pre and post course assessment and confidence ratings*	• Cohort 1 trained using the palliative care toolkit - March and June 2011 • Cohort 2 trained January and March 2012	• Cohort 1 – 11 nurses completed pre and post course assessment and confidence ratings • Cohort 2 – 16 nurses completed pre and post course assessment and confidence ratings, 1 only completed the pre and not the post-course assessment	• Total of 25 participants completed pre and post course assessment and confidence ratings	• March 2011 – March 2012
2. *Course evaluation forms*	• Cohort 1 trained using the palliative care toolkit - March and June 2011 • Cohort 2 trained January and March 2012	• Cohort 1 – 11 nurses trained and completed evaluation forms *[check this]* • Cohort 2 – 16 nurses trained and completed evaluation forms	• Total of 27 participants completed evaluation forms	• March 2011 – March 2012
3. *Audit re the availability of the clinical guidelines*	• 11 clinical guidelines developed and distributed to the link-nurses for use on their wards	• Audit carried out across 26 link nurses and the wards where they were working. (1 link-nurse no longer working at the hospital)	• Total of 14 wards audited	• Clinical guidelines developed between June 2011 and July 2012 and distributed to the link-nurses. • Audit carried out of availability of clinical guidelines in [Insert date]
4. *Review of activity database from the link-nurses and MPCU*	• Database developed and revised 2011–2013 to include all activity from link-nurses and MPCU	• Data from MPCU team and 26 link-nurses (1 nurse having retired)	• Activity for 2829 patients reviewed	• Data reviewed from July 2012 - December 2013
5. *Review of action plans*	• Cohort 1 action plans developed June 2011 • Cohort 2 action plans developed March 2012 • Both cohorts reviewed and revised September 2012 • Action plans reviewed regularly by link-nurse co-ordinator	• Cohort 1 – Action plans submitted by 11 nurses • Cohort 2 – Action plans submitted by 12 nurses	• Action plans reviewed from 26 nurses, as 1 was no longer working at Mulago Hospital	• 2011-2013
*6. Focus Group Discussions*	• 4 FGDs held	• 13 Link-nurses, including nursing management • 7 MPCU team members, including the co-ordinator of the link-nurse programme	• Total of 20 participants	• FGDS carried out between May and August 2013
*7. Most Significant Change*	• MSC stories completed by participants in [date]	• 16 (?check that) nurses submitted MSC stories	• Following review, 4 stories were identified as most significant for the impact on self, 4 on patients, and 2 on colleagues	• September 2013 – May 2013

### Data collection

The evaluation was overseen by JD (Honorary Professor of Nursing) and data was collected by the MPCU research nurse (MB) and M&E Manager (GK). The research team were female, and all participants apart from one doctor from MPCU were also female. The research team have all been trained in palliative care research and MB and GK both attended an advanced research school in 2012. Whilst part of the MPCU, neither MB or GK had been key to the implementation of the link-nurse programme, although they may have been known to the link-nurses as MPCU members. A semi-structured focus group guide was developed and utilised. FGDs were held at Mulago Hospital, lasted for an average of 40 min, conducted in English, and recorded digitally.

Information sheets were provided to all participants and consent was gained prior to commencement of all FGDs. It was explained to participants that participation in the study was voluntary, it would not affect their role in the link-nurse programme, and they were free to withdraw from the evaluation at any time. Of the 27 trained link-nurses and nine team members of the MPCU team, 14 nurses and seven team members participated. The team members who did not participate (*n* = 2) were involved in the evaluation and therefore excluded. The nurses who were unable to participate (*n* = 13) had moved on from their wards, were on different shifts or on leave. Only the evaluators and participants were present for the FGDs.

Quantitative data including pre and post course assessments and course evaluations was collected as part of the link- nurse programme, commencing in 2011, and the MPCU database is updated regularly and results were reviewed December 2013.

### Data analysis

Using an adapted method developed by Corbin and Strauss [29], data was analysed using thematic analysis, where the transcripts of data was reviewed, themes and patterns identified that appeared repeatedly or those that were significant outliers sitting in contrast to the norms. FGDs were transcribed and the transcriptions compared with the digital recordings for accuracy. Codes were generated from the text, drawing out themes and examples pertaining to the evaluation. The issue of data saturation did not impact the number of FGDs undertaken due to the finite number of link-nurses available. The coding framework was developed by JD and MB and data coded by MB and checked by JD. Coding was undertaken manually without the use of software. As per MSC methodology, [30] MSC stories were reviewed by a team and the most significant stories identified and agreed upon. Quantitative data was analysed using SPSS Version 22. The overall results of the evaluation were reviewed by the MPCU team.

## Results

Twenty-seven link-nurses were trained during the project, eleven in cohort 1 and 16 in cohort 2. Their backgrounds varied but included qualified nurses from all of the key wards where individuals with life-threatening conditions were admitted, and were selected in conjunction with the senior Nursing Administration at the hospital. All 27 nurses participated in the evaluation through their pre and post course assessment results, their training evaluation forms, action plans and activity logs. Sixteen wards were audited for access to clinical protocols, covering 25 link-nurses. Twenty participants took part in the FGDs, 13 of the link-nurses including one from senior management and seven members of the MPCU team, including the link-nurse co-ordinator. A summary of data collection and participants can be found in Table 2.

Three key areas were reviewed in the analysis: the training and link-nurse activity, which report the results of the quantitative analysis and then the findings from the qualitative analysis which are presented in five themes.

### The training

**Fig. 1:**
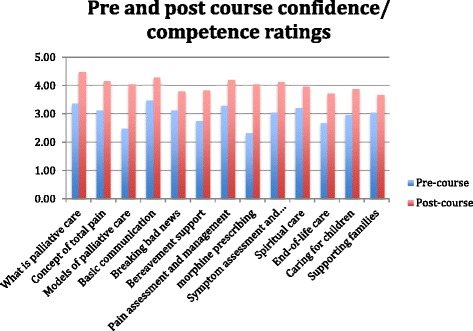
Mean pre and post course competence/confidence scores

Prior to the start of the training participants were asked to complete a pre-course confidence rating. Participants were asked how confident/competent they felt on a scale of 1–5 with 1 being ‘none’ and 5 being ‘excellent’ with regards to the palliative care competencies being addressed in the training. All participants completed the pre-course questionnaire (*n* = 27), and 25 completed the post-course questionnaire (*n* = 25). Pre-course confidence varied across the different areas (Fig. 1), with participants being least confident in morphine prescribing (mean = 2.32), models of palliative care (mean = 2.48), end-of-life care (mean = 2.68) and bereavement support (mean = 2.76), and most confident in basic communication (mean = 3.48), what is palliative care (mean = 3.36) and pain management (mean = 3.29). Significant increase was seen in confidence/competence following the training, with mean scores in all areas showing significant improvement (*p* < 0.001 for ten variables, and *p* < 0.01 for three variables (Table 3). The greatest improvements were seen in the areas of morphine prescribing (increase in mean of 1.72), models of palliative care provision (increase in mean of 1.56) and what is palliative care (increase of mean of 1.12).

**Table 3:** Paired *t*-test significance testing for pre and post test scores

Pair	Question	*N*	Mean	*t*-test (*p* value)
1	What is palliative care?			
	• Pre-course • Post-course	25	3.36 4.48	.000
2	Concept of total pain?			
	• Pre-course • Post-course	25	3.12 4.16	.000
3	Models of palliative care provision			
	• Pre-course • Post-course	25	2.48 4.04	.000
4	Basic communication			
	• Pre-course • Post-course	25	3.48 4.28	.000
5	Breaking bad news			
	• Pre-course • Post-course	25	3.12 3.80	.004
6	Bereavement support			
	• Pre-course • Post-course	25	2.76 3.84	.000
7	Pain assessment and management			
	• Pre-course • Post-course	24	3.29 4.21	.000
8	Morphine prescribing			
	• Pre-course • Post-course	25	2.32 4.04	.000
9	Symptom assessment and management			
	• Pre-course • Post-course	25	3.04 4.12	.000
12	Spiritual care			
	• Pre-course • Post-course	25	3.20 3.96	.001
13	End of life care			
	• Pre-course • Post-course	25	2.68 3.72	.000
14	Caring for children			
	• Pre-course • Post-course	25	2.96 3.88	.000
15	Supporting Families			
	• Pre-course • Post-course	25	3.04 3.68	.007

Throughout the evaluation, the nurses noted that all of the topics had been very useful, and where the were topics were already familiar, revision of those topics had been helpful. Participants felt the course should be made available to more nurses within the hospital and the number of days of the training increased. Nurses also spoke of the importance of including patients as part of the training process in order for the patient voice to be heard. It was noted that this was done during the mentorship and supervision at the bedside. Nurses appreciated the interactive nature of the training, along with the use of case discussions and scenarios.

Key issues the nurses said they would take back to their practice were assessing patients in a holistic manner, encouraging others to be empathetic and to communicate with patients and their families the ‘truth about the sickness’. Working with colleagues and sharing what they had learnt was identified as important in order to change attitudes for palliative care and to enable them to practice their skills on their wards. Working hand in hand with the palliative care team was also seen to be important, and the training had helped in building relationships with the team.

Challenges that the nurses had experienced in implementing palliative care between the two taught aspects of the course included working with others, in particular the doctors for pain and symptom management, the overwhelming number of patients needing palliative care, issues of availability of medications and radiotherapy (frequent technical delays due to only one elderly cobalt machine), time management and meeting holistic needs.

### Link-nurse activity

The activity of the link nurse was used as an indicator of the effectiveness of the cadre and their capacity to engage in the work to which they had been trained. Throughout the duration of the evaluation each link nurse submitted monthly activity sheets, and data was entered into the MPCU database. Activity sheet information included patient name, diagnosis, interventions given, and the reason for referral when referred to MPCU.

**Table 4:** Patient categorisation system

Category 1:	Patients with generalist palliative care needs that can be met at ward level by all health and social care workers
Category 2:	Patients with generalist palliative care needs that can be met at ward level by health care workers who have had additional training e.g. link-nurses.
Category 3:	Patients with complex palliative care needs requiring referral and input from one member of the specialist palliative care team
Category 4:	Patients with complex palliative care needs requiring referral and input from multiple members of the specialist palliative care team.

The database was reviewed from July 2012 to December 2013. During that time 2447 patients were identified by the link nurses as having palliative care needs. Two thousand one hundred thirteen of these (86 %) were managed by the link nurses on the wards, and 334 (14 %) of these were managed by the MPCU. A further 382 patients were referred to MPCU from other sources. Thus during that period, 2829 patients with palliative care needs were cared for in Mulago Hospital, 2113 (75 %) were cared for by the Link-nurses and therefore seen to have generalist palliative care needs (Categories 1 and 2) and 716 (25 %) were cared for by the MPCU and therefore seen to have specialist palliative care needs (Categories 3 and 4) - see Table 4.

**Fig. 2:**
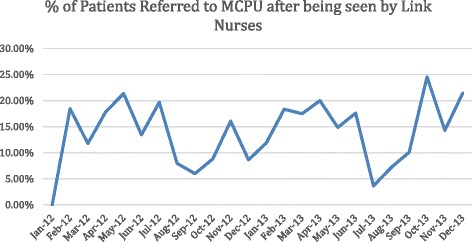
Percentage of palliative care patients referred by the link nurses January 2012 – December 2013

**Fig. 3:**
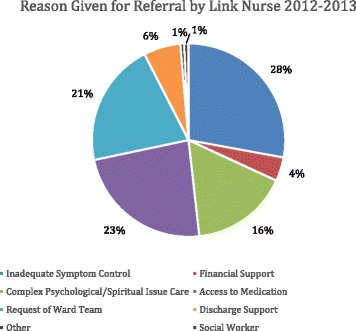
Reasons given for referral by the link nurse during 2012 and 2013

Referral to the MPCU varied across the year, with referrals depending on various factors including how busy the hospital was, the availability of link-nurses (e.g. holidays, night duties etc.) and the availability of the MPCU, and ranged between 10 and 25 % (Fig. 2). Reasons for referral included inadequate symptom control (28 %), access to medications (23 %), the request of the ward team (21 %), and complex psychological/spiritual needs (16 %) (Fig. 3).

Eleven clinical guidelines and protocols were developed and trialled by the MPCU team. They were adopted by the Ministry of Health and distributed around the hospital via the link nurses. The link nurses were encouraged to use the protocols and to share them with colleagues on their wards. The protocols covered constipation, delirium, dyspnoea, end of life care (*x*2), fungating wounds, mouth care, nausea and vomiting, pain management in adults and in children, and spinal cord compression. These guidelines were produced as a laminated A4 sized set. The protocols for pain management in adults and in children, were also hung up as posters in the wards. An audit of the 14 link nurse wards ascertained that the poster was displayed on 13 wards (93 %). In the one ward without the poster displayed the link nurse had moved to theatres and had displayed the poster there instead. The clinical protocols were available on the wards for use on seven out of the original 14 wards (50 %), The protocols had also been taken to the theatre block from the original ward. On one of the other wards only the clinical protocol on pain in children was available and being used. On the remaining five wards (36 %) reasons for the protocols not being available included not knowing where they are, and the link nurse having them at home.

Action plans were completed by all of the link-nurses on the last day of their training programmes. These were reviewed monthly by the link-nurse co-ordinator and an appraisal of the action plans was carried out. The nurses had identified similar activities on their individual action plans, which included: sharing new knowledge with the ward team; formally reporting on the training programme to nursing management; integrating palliative care into their daily work; completing the activity logs, collaborating with the MPCU, and conducting new teaching sessions on palliative care in the wards. The majority of planned activities had been undertaken, but more support and resource capacity was required for conducting training sessions on the wards for staff. The MPCU team offered to help run these training sessions and so several were organised jointly with the MPCU team on the different wards, and these are ongoing.

### Main themes identified from the FGDs and MSC stories

Four FGDs were held with link nurses and members of the MPCU. Link-nurses were also encouraged to submit Most Significant Change Stories. These stories were reviewed by the MPCU team and key stories identified in each of the three areas – impact on the individual, the patient and the organisation. Themes identified from both the FGDs and the MSC stories are discussed.

#### Change in attitude

For many of the link nurses, the programme has resulted in changes in their attitudes towards both patients and palliative care. Palliative care patients are now seen as integral to their work on the ward and they realise that there is always something that can be done for them:

*“The training has helped me a lot and I think excellence is not a skill it is an attitude. Me whenever I could see the word palliative care, I used to think that the patient has few hours, I didn’t know that people can live, the first patient I saw up to now is still living. My attitude changed a lot, palliative care I thought whoever is for palliative care, death sentence, but at least now I know, someone can be there.”* (LN-11)

Specific changes in attitude were seen in the management of pain and the use of morphine:

*“I used to think when the patient is given morphine, they become addicted to it so up to now I still continue to tell my colleagues that when they write morphine we give it accordingly it ask the patient, there are times when the morphine is finished or has not got it he does not feel the pain but for you know it has to be.”* (LN-12)

Several of the MSC stories illustrated these changes in attitude, both generally and for pain management:

*“since I trained in palliative care there is a great change in me. After the successful completion of the course I was equipped with better and modern palliative care knowledge that is required in the management of cancer multi modernity approach especially those patients in pain. I always make sure they are attended to immediately without delay. This course put me in a better position to teach and supervise the students and the way of handling patients changed as I said especially in pain.”* (MSC-12)

#### Developing new skills and knowledge

Key to the impact of the link-nurse programme has been the development of new skills and knowledge of palliative care by the link-nurses. It was this knowledge and skills that has helped them to improve the care that they are giving:

*“What I can say I’ve utilised it in improving the care I’m providing on patients initially I didn’t know much about PC I would see a patient needs some special care but actually I would not come out to say which care exactly, whether the pain needs psychosocial support. Much as we do it in our training but I think somehow it has not been so practical but now I’m improving.”* (LN-10)

Along with being able to improve care, the nurses recognised that there are times when they are unable to provide the care and need the support of the MPCU:

*“one could be failure to respond to morphine however big the dose could be and may be some patient may complain that they get continuous constipation to the extent that the patient may refuse taking morphine so those are some of the challenges whereby you call in the supervisor to see what to do and them may be other carer’s my be like social problems where by the care taker is in disagreement with the patient to the extent that the care taker may threaten to run away and really you may have talked to the care taker and the counselling may not have taken effect. So you may really have to act upon the supervisor immediately.”* (LN-5)

Areas where the nurses felt they had developed new knowledge and skills included pain management:

*“For me, before I did this course, mostly let me talk about pain, I didn’t know how to manage pain of a patient when a patient comes and tells me that he has pain, I could just focus on that pain but since I did this course, I can control pain. I don’t know how I can say it.”* (LN-13)

Communication:

*“It also helped me in breaking the bad news. Before they could not tell the patient or the care taker about the condition but now days its different. You try to dodge you keep on asking but now I can always tell them about the diagnosis then counsel them about the care and the outcome.*” (LN-5)

Confidence:

*“For me it has built my confidence in the care of patients with HIV with pain and I can now comfort ability give pain analgesic drugs like oral morphine I don’t have to wait for the doctor to start managing pain.”* (LN-6)

Several of the MSC stories illustrated this change in knowledge and skills through patient stories:

*“Patients are happy and appreciate our efforts they feel they are loved and cared for by the palliative care team and really feel the worthiness of living through our management. One of my patients told me that he is really appreciated because he had feared to be done a gastrostomy but as a team he was counselled and later accepted. He came to believe that actually without the gastrostomy he would be dead by now. Not by the cancer but by starvation. He has gained weight and feels strong. He is now able to visit his business settings, has ably made a will and at least accomplished some of his unfinished business.”* (MSC-11)

#### Relationships

The link nurse training helped nurses both build stronger relationships and also analyse the impact and effect of relationship building as a tool within holistic palliative care:

*“I think it has created that bond between the link nurse and the patient because these patients have a lot of confidence in us when we help them, the way we talk to them.”* (LN-2)

*“And that quite a dramatic change from a situation where morphine was not being accessed at ward level..…. So they can’t expect to do that on their own ……. so its about the relationship say the good relationship on the wards and empowering them”* (MPCU-2)

The nurses and the MPCU reported feeling that that they were working better together and collaborating in the care of individuals with each other and other members of the ward team.

*“In the training what was that was it about team work what was it about that has helped you to work as not in parallel. You learnt to appreciate that patient care you can’t do it alone as an individual nurse.”* (LN-1)

*“Partly the implementation has been by them and sometimes they may be sometimes people can comment but they come on our ward rounds which is brilliant and we can jointly discuss the management and follow up say can happen by the link nurses and maybe I just want to say to mention the seniors advocacy, you have mentioned advocacy at ward level and the team will smile because one of the quotes usually used was from the Senior nurse in MOH. We are all PC when the PC team used to come to my ward I would think they are doing their work but now I realise its our work that sense of people owning PC and nurses and doctors starting from the ward beginning to say we can provide and seeing PC is part and parcel of clinical care*.” (MPCU-2)

An MSC story captured the increased value of relationships between the nurses and patients, and nurses and colleagues:

*“I nursed a patient who did not have anybody to assist her either physically or financially. As a palliative care nurse, that patient became more than a patient but a friend such that even when I was not officially on duty I would pass by to check if she has bathed or has had something for the stomach. My colleague was so amazed about my actions that she was touched and whenever I was not there, she filled the gap. When the patient died, together we made sure we look for any contacts of her relatives to take her body for burial at her ancestral grounds. Thank God our efforts were successful and from that day on my colleague, who has had no training in palliative care learnt from me that there is always something we can do.”* (MSC-14)

#### Increase in access to and strengthening of palliative care

The numbers of patients referred to MPCU increased over the two years:

*“Yes it helps with earlier identification of patients so like previously as before the programme you would find patients sent to us…….. but after training the link nurses they have identified patients with PC needs and referred them early so we are finding that patients are seen earlier than before.”* (MPCU-5)

Transfer of knowledge on palliative care from the link-nurses to other colleagues appears to have increased accessibility to palliative care throughout the hospital:

*“My colleagues are happy and they have appreciated palliative care. In that they find out that those who had the training have some skills that they don’t have. Secondly many of them who are training come for help to answer some questions on palliative care. Nurses are getting on well with four hourly morphine and that it was working well other than what they knew before being a drug of addiction and had bad effects. Many of them inquire as to when the next training would be. Meaning they are willing to get this knowledge to help the patients better.”* (MSC- 15)

*“In Radiotherapy I can also tell you a scenario, we had a patient with cancer breast, this patient this lady had a fungating wound and the children were boys these boys wanted to, the mother to be admitted somewhere because nobody could dress that wound, but I sacrificed and told those boys I would do it and I did it cleaned it and the lady was in pain. I gave diclofenac to her she took it I dressed the wound well and the pain went, the boys were so happy, the patient was from north.”* (LN-13)

There was a deepening sense of value and work-worth among nurse link practitioners, along with an increase in visibility, recognition and ownership of palliative care in the hospital.

“*on our ward the clinical head was appreciative he said since we started the palliative care work on the ward patient are no longer yelling of pain and the patients are manageable. He is happy about it and I am happy because some of us give in time to stay with those patients when the other people go off duty but you have or give extra time.”* (LN-8)

*“Ya when you are going to do the training which people to chose because we see (exactly) it has been very good (ownership) and in fact as we know one of the senior nurse managers actually came on the training and she said if you are going to train my nurses I need to know what you are training them on and came on the training.”* (MPCU-2)

The link-nurse programme has had a positive impact on care and strengthening palliative care at Mulago Hospital”:

*“Absolutely, you are talking creatively we are talking I feel maybe I’m biased………… this idea of developed link nurses but it can only be part of strengthening health systems approach which needs to involve both the policy makers those who control things like deployment as we discussed as well as the individual personal cadres.”* (MPCU-2)

#### Challenges

The link-nurses experienced many challenges in trying to integrate palliative care into their daily practice. Challenges relating to clinical practice in particular included issues around the use of morphine. Although the link nurses were comfortable to recommend morphine for managing pain, other health professionals on the wards, such as the prescribing doctors, on whom the nurses rely for prescribing, may not be:

*“My main challenge is on morphine prescriptions. Our paediatricians its not easy to convince to talk to a paediatrician and convince him to prescribe morphine to a child and if they do so they only give start dose they find problems convincing them.”* (LN-3)

Even when the doctor does prescribe they also sometimes experience challenges with the lack of availability through stock outs on the ward:

*“I think there should be constant supply of morphine because at time there is shortage of supply in the hospital you look at someone, that person doesn’t have morphine. In agony and there is nothing much you can really do you can’t go to the open market and by … and somebody is pain.”* (LN-1)

Whilst education is important and the link-nurses have recognised the need for training their colleagues, their lack of interest in palliative care can be challenging:

*“It could also be interest you know this PC whatever care it takes heart. You don’t just tell somebody that come you know you see we want to have a meeting and talk about this care not all. Even when you call for a CME but you find that you are ten people on the ward and only two have come so you mean not many nurses have a kind heart?”* (LN-5)

The MPCU team commented on the challenges of the link-nurses referring patients to them, and whether they were referring enough patients to them:

*“…… but there is a question mark on the number of patients that have been referred ….. because I know the form has been adjusted several times its like it was adjusted once.”* (MPCU-3)

Whilst the link-nurses appreciated the training challenges were experienced with regards to the length of the training and the mentorship and supervision as they were often not available when the link-nurse co-ordinator visited them:

*“its like understanding everything during that short period”* (LN-4)

## Discussion

The introduction of the link-nurse programme at Mulago Hospital, UHI and UCI has extended the provision of palliative care within the hospital. The number of patients receiving palliative care increased from 611 during 2011 to 1788 in 2013. Between July 2102 and December 2013, the link-nurses cared for approximately 75 % of those patients needing palliative care within the hospital. The remaining 25 % with more complex problems were referred to the specialist palliative care team, allowing them to focus on patients who needed their expertise.

The programme demonstrates that nurses working in the wards of busy hospitals in sub-Saharan Africa can be trained and supported to provide generalist palliative care to patients, working with and referring to, the specialist palliative care team as needed and working as a focal point for the core clinical teams. Thus whilst the cultural context and amount of resources may differ, this is similar to some of the earlier studies done in the UK which suggests that link nurses had increased knowledge and skills, increased confidence and had an influence on the provision of palliative care in hospital wards [19, 21–23]. Key to the success of the link nurse training in Mulago has been training and ongoing mentorship and supervision, an important aspect identified in previous studies [19, 22] along with ‘ownership’ by the nursing administration. This last aspect of ‘ownership’ is key to the ongoing sustainability of the programme, and both the link-nurses and members of the nursing management are now seeing palliative care as ‘their work’ rather than that of the MPCU only. The link-nurse programme was not however without its challenges, and whilst some of these, such as the availability and prescription of medications such as oral morphine, may be context specific, others such as the availability of education, and being release from the ward to attend training sessions are challenges that have been found in other contexts as well [21–23].

During the the period of this evaluation training programmes were also developed to support postgraduate medical competencies for palliative care and ongoing integration into the undergraduate nursing and medical curriculums. This programme was also developed in the context of national and regional leadership for palliative care in Uganda, ongoing advocacy and awareness, opportunities for specialist Diploma and Degree level training and significant partnerships including the Ministry of Health, the national association (PCAU) and key stakeholders. Thus developing education programmes for all levels of care as identified in the WHA resolution on palliative care [8].

Hospital-based palliative care teams [31] are a recognised model of palliative care provision, although they are less common in Africa [6]. Such teams, as well as providing clinical care are seen as resources to ward-based hospital staff in the improvement of palliative care provision [23] as manpower and resource issues make it impossible, and inappropriate, for specialist palliative care services, such as that provided by MPCU, to be involved in providing palliative care to everyone throughout the hospital [32]. The unmet need for quality palliative care within hospitals, and within the government health service is great, and so models need to be developed and adapted that will meet these needs and support referral and discharge pathways. National hospitals also provide important leadership, research and training hubs. The integration of palliative care into health systems at all levels is key to the ongoing development of palliative care within the region, and for ongoing sustainability of services. Whilst much good palliative care provision is provided by specialists through non-governmental and faith based organisations, integration into the government is the only way to make palliative care provision universal, hence recent emphasis on an integrated response to palliative care through national health systems [33–35]. A study in Malawi, [36] along with the needs assessment carried out at Mulago Hospital [11] identified the need for integrated services, recognising that such services are effective in addressing patients needs. The numbers needing palliative care in hospitals such as Mulago Hospital are unlikely to decrease. Alongside those patients patients and families with complex problems requiring specialist support, there will also be a significant proportion who have generalist needs with a variety of diagnosis. In settings where almost half of the inpatient population have palliative care needs, models of care that are not solely reliant on a small specialist resource and which integrate and empower all health care workers are needed [22, 37, 38].

### Limitations of the evaluation

Data was collected by the MPCU team who were not directly involved in the programme, but as they were part of the team, this could have been seen as a source of bias. The FGDs were held at set times and the busy wards and challenged workforce crises meant that some nurses were not on duty or were not able to be released for the FGDs. It could be argued that those who were not available were not as committed as those who made themselves available thus introducing bias to the results, although this is unlikely. Other limitations are similar to those found in other studies [19, 22, 23] including the lack of patient participation, a lack of reviewing outcomes of care and also not including other health workers such as ward managed and doctors working alongside the link nurses.

### Recommendations

For Mulago Hospital, UHI and UCI:

- Ongoing support for the link-nurse programme, including retention of the link-nurse co-ordinator.
- Extending training of link-nurses within the hospital to enable full coverage.
- Including the link-nurse programme as part of comprehensive, integrated palliative care training for all cadres of health and social workers.
- Review of referral patterns from the link-nurses to the MPCU to ensure that the specialist team are seeing the most relevant patients.
- Review of the categorisation system to ensure that complex needs are being recognised.
- Ongoing quality evaluation of the palliative care provision to ensure improved patient and family experience.

For Uganda and the region:

- To undertake needs assessments in other hospitals, both at the regional and district levels to ascertain the level of need for palliative care, and the appropriate model of care.
- To expand the integrated model of care including the link-nurse programme to other hospitals, and cadres, in Uganda.
- To expand the integrated model of care including the link-nurse programme to other hospitals in the region e.g. in Rwanda, Kenya, Zambia etc., and review the model and its international transferability.
- To gain recognition and ownership of the programme from the Ministry of Health, to aid recognition and performance related benefits of link-nurses once trained.

## Conclusion

The link-nurse programme has been successful in meeting its aims of equipping nurses from different wards with knowledge and skills enabling them to provide generalist palliative care alongside their clinical team and improving links between the wards and the MPCU. It has radically improved palliative care within Mulago Hospital, UHI and UCI both in reaching more people, but also in providing care according to extent of needs, enabling specialist palliative care practitioners to concentrate more on those with complex needs, as well as offering training and mentorship. Palliative care need in hospitals such as Mulago Hospital are great, and integrated models of care that strengthen health systems need to be developed in order to meet that need and to ensure that all patients in the hospitals who need it have access to palliative care services, through both generalist and specialist provision. Further work is needed to test this model in smaller hospitals, and to develop a model for integrated care in health centres and dispensaries, and linking these settings together in a whole systems approach to ensure universal coverage of palliative care provision.

### Ethics and consent to participate

Ethical approval was gained from both the Mulago Hospital Research and Ethics Committee (MREC 312) and the Uganda National Council for Science and Technology (UNCST SS3118). Data was transcribed, anonymised and stored in a secure password-protected computer.

We confirm that within the consent forms we obtained consent to publish from the participants to report individual annonymised data.

### Availability of data and materials

Annonymised data is held at MPCU. It is not possible to share this data due to regulations stated in the ethical approval.

## Abbreviations

COREQ: COnsolidated criteria for REporting Qualitative research
FGDs: Focus Group Discussions
LN: link nurse
MPCU: Makerere/Mulago Palliative Care Unit
MREC: Mulago Research and Ethics Committee
MSC: most significant change
PC: palliative care
SPSS: Statistical Package for Social Scientists
UNCST: Uganda National Council for Science and Technology
WHA: World Health Assembly
